# Gender differences in adolescents’ perceptions toward dentists using the Japanese version of the dental beliefs survey: a cross-sectional survey

**DOI:** 10.1186/s12903-019-0845-y

**Published:** 2019-07-12

**Authors:** Hiroyuki Karibe, Yuichi Kato, Kisaki Shimazu, Ayuko Okamoto, Masahiro Heima

**Affiliations:** 1 Department of Pediatric Dentistry, School of Life Dentistry at Tokyo, Nippon Dental University, 1-9-20 Fujimi Chiyoda, Tokyo, 102-8159 Japan; 2 Department of Pediatric Dentistry, School of Dental Medicine, Case Western Reserve University, 10900 Euclid Avenue, Cleveland, OH 44106-4905 USA

**Keywords:** Dental fear, Adolescents, Confidence in dentists, Anxiety, Gender difference

## Abstract

**Background:**

While adult women show greater dental anxiety than adult men, few studies have examined gender differences in adolescent perceptions of dentists. Therefore, this cross-sectional study aimed to evaluate the gender differences in adolescents’ perceptions toward dentists by using the Japanese version of the Dental Beliefs Survey (DBS) and the factor structure of the DBS.

**Methods:**

We conducted surveys at schools, and 957 Japanese adolescents (403 girls and 554 boys, aged between 13 and 15 years) participated in this study. To assess their confidence in dentists, participants were asked to complete the self-reported, 15-item Japanese version of the DBS. We performed a Welch’s *t*-test and a one-way analysis of variance to assess differences in DBS scores by gender and age. Factor analysis (principal components, varimax rotation) was used to assess the scale’s factor structure.

**Results:**

A significant gender difference was observed in the DBS scores (*P* = 0.018), suggesting that boys exhibit greater negative perceptions toward the behavior of dentists than girls. However, there was no significant difference found among ages. The factor analysis yielded two results: Factor 1, “trust” (seven items); and Factor 2, “lack of control” (five items). Notably, the factor structure differed according to gender. As such, by including only factors with eigenvalues above 1.0, the DBS for girls comprised “trust” (seven items) and “communication” (three items), while that for boys comprised “lack of control” (six items) and “belittlement” (six items).

**Conclusions:**

This study identified two factors of differing strengths pertaining to the confidence of Japanese adolescents in dentists. Gender differences in perceptions toward dentists were observed. Accounting for these differences may improve the effectiveness of strategies to lower dental anxiety and foster positive dental beliefs in young patients.

## Background

In young patients, distrust of dentists may often result in dental fear [1]. Negative beliefs about dentists have been shown to have a strong relation to the high frequency of cancellations and missed appointments [2]. Another study reported the role of psychological variables—such as being embarrassed by dental fear—that lead to avoidance, deterioration in dental health, and feelings of shame that culminate in reinforced avoidance [3]. However, little attention has been directed toward the subjective perceptions of adolescents regarding the behavior of their dentist.

Beliefs about dentists have been assessed by a self-reported questionnaire—the Dental Beliefs Survey (DBS)—developed by Milgrom [4]. The purpose of the DBS is to identify to what degree the patient perceives the behavior of the dental professional as contributing to his or her fear or anxiety, and the information obtained from the DBS is useful from both diagnostic and prescriptive standpoints. The survey questions are designed to help dental professionals tailor their approach to best address the specific concern of the patient [4], so, in that way, it is more useful than a standard fear or anxiety questionnaire as it is more focused on the effects from the dentist’s specific behavior.

Several international studies using the DBS have been conducted in various populations [5–7]. While Milgrom [4] suggested that the original DBS fit a four-factor structure, only a few studies have been conducted to assess its structure in different populations [8]. To the best of our knowledge, no epidemiological studies have been conducted to evaluate Japanese adolescents’ confidence in dentists. Further, no study has assessed the factor structure of DBS results of the Japanese adolescent population. Thus, a study accessing dental beliefs in Japanese-speaking adolescents is required.

Although adult women show greater dental anxiety than adult men [9, 10], evidence regarding the differences in dental fear between boys and girls has been inconsistent. Some studies have reported that girls are more fearful [11, 12], while others have indicated no significant difference between boys and girls [13, 14]. Schienle et al. [15] studied gender difference in neural correlates of dental phobia. They indicated that compared to male individuals with dental phobia, female individuals experience less cognitive control and show more avoidance behavior during treatment. Another study showed that, after the completion of the treatment, women remembered more pain and other negative experiences than men [16]. Considering the gender differences in perceptions toward dentists may improve the effectiveness of strategies for lowering dental anxiety in young patients.

As such, this study aims to evaluate the confidence in dentists of Japanese-speaking adolescents using a Japanese translation of the DBS, examine the factor structure of the Japanese version of the DBS, and assess the gender differences. We expect girls to report more negative perceptions of dentists than boys and for their factor structure of the DBS to differ from that of boys.

## Methods

### Participants

The participants of this study, individuals aged 13–15 years, were also participants in a school-based cross-sectional survey regarding temporomandibular disorders [17]. The present study included Japanese adolescents (13–15 years old) from a regional survey of 998 students from three junior high schools in Suginami, Tokyo. From the 23 junior high schools that we had approached, the administration of these three schools consented to participate in this study. No schools with intellectually disabled or learning-disabled students were included.

Participation in this survey was voluntary and anonymous. A questionnaire concerning perceptions toward dentists was distributed in class; the students were asked to respond to the questionnaire only if they were willing to complete the survey. Junior high schools in Japan are legally obligated to conduct annual oral checkups for school students. All data were collected during the schools’ annual oral checkups, held between October and November 2011 at all three schools.

Among the 998 students who participated in this study, 41 students missed one or more questionnaire items, and hence their data were excluded. Therefore, the final sample included data of 957 students (Table 1). This sample could be representative of the 10,100 students aged between 13 and 15 who attended junior high schools in Suginami during 2011.

**Table 1:** Number of participants, age, and gender distribution in the study sample

Age (years)	N	Girls (%)	Boys (%)
13	291	117 (40.2)	174 (59.8)
14	325	142 (43.7)	183 (56.3)
15	341	144 (42.4)	197 (57.6)
Total	957	403 (42.1)	554 (57.9)

The study protocol was reviewed and approved by both the Ethics Committee at the Nippon Dental University School of Life Dentistry (NDU-T2011–21) and the local education authority and conformed to the guidelines of the Declaration of Helsinki. The students and their parents provided their informed consent prior to participation.

### Measures

The DBS consists of 15 items with a 5-point Likert scale (1: never, 2: a little, 3: somewhat, 4: often, 5: always) with scores ranging from 15 to 75, where 75 indicates maximal negative perception toward dentists. The DBS measures the subjective perceptions regarding the behavior of dentists and the way in which dental care is delivered; it also takes the aspect of control into account (Table 2). Milgrom et al. [4] noted four separate dimensions of the DBS: “communication,” “belittlement,” “lack of control,” and “trust.” Five questions (1, 3, 4, 14, and 15) in the survey represent the dimension of “communication,” which explores how well the patient thinks the dentist communicates. Three questions (6, 9, and 10) represent the dimension “belittlement,” which examines the respondent’s anticipation of how the dentist might view their fear. Three questions (5, 12, and 13) represent the dimension “lack of control,” which examines the respondents’ beliefs regarding their ability to control the situation while undergoing treatment. Finally, two questions (7 and 8) represent the dimension of “trust,” which examines how skeptical the patient is of the dentist. Two questions (2 and 11) are not included in these four dimensions of the DBS; these questions concern fear that the dentist will not perform well due to other factors.

**Table 2:** Original version of the Dental Beliefs Survey

	Never	A little	Some-what	Often	Nearly always
1. Dentists do not like patient requests.	1	2	3	4	5
2. Dentists seem to be in a hurry, so I feel rushed.	1	2	3	4	5
3. Dentists do not provide clear explanations.	1	2	3	4	5
4. Dentists do not really listen to what I say.	1	2	3	4	5
5. Dentists do what they want, no matter what I say.	1	2	3	4	5
6. Dentists make me feel guilty about how I care for my teeth.	1	2	3	4	5
7. I am not sure I can believe what the dentist says about the dental work that is needed.	1	2	3	4	5
8. Dentists say things to try and fool me.	1	2	3	4	5
9. Dentists do not take my worries seriously.	1	2	3	4	5
10. Dentists make light of my fears.	1	2	3	4	5
11. I worry whether dentists do a good quality job.	1	2	3	4	5
12. If it hurts, I do not think the dentist will stop.	1	2	3	4	5
13. I do not feel I can stop for a rest during treatment.	1	2	3	4	5
14. I do not feel comfortable asking questions.	1	2	3	4	5
15. The thought of “hearing bad news” could be enough to keep me from going for treatment.	1	2	3	4	5

To assess their confidence in dentists, participants were asked to complete the self-reported, 15-item Japanese version of the DBS. The questionnaire was originally developed in English, but, as Japanese was the common language among all participants, the questionnaire was translated into plain Japanese by the authors. To confirm that the English and Japanese questionnaires had the same content, the initial Japanese translation was retranslated into English by bilingual faculty members, and the contents of the original English and retranslated English versions were compared to ensure consistency. All versions were also analyzed and compared by the authors, and a final version was obtained.

Additionally, to assess the equivalency of the translation, a preliminary study was conducted. The questionnaire was given to eight bilingual adult volunteers, twice under similar conditions. Each volunteer was randomly assigned one version of the DBS, either English or Japanese, and asked to complete it. The volunteers were then asked to complete the other version of the DBS on the following day and under similar conditions, without referring to the previous questionnaire. The Kappa coefficient was used to evaluate the equivalency of the language. Accordingly, 14 of the 15 items on the questionnaire obtained an average Kappa value of 0.61—with two items obtaining a score equal to 1, while others had a Kappa value between 0.40 and 0.77. While it was not possible to calculate the Kappa coefficient for one of the 15 items, seven of the eight volunteers responded with the same answers regarding this item in both versions. These results indicated good equivalency between the two versions of the questionnaire.

To determine the repeatability of the Japanese version of the DBS, another preliminary study was conducted. The questionnaire was administered to 12 adult volunteers who did not participate in the former preliminary study, twice under similar conditions. The volunteers were asked to complete the Japanese version of the DBS questionnaire, and, a week later, they were asked to complete the same questionnaire under similar conditions, without referring to the previous one. We calculated the intraclass correlation coefficient (ICC), in which subjects and occasions were considered random factors [18]. The average ICC for all questionnaire items was 0.93, indicating that the repeatability of the questionnaire was sound. (A copy of the Japanese version of the DBS is available from the corresponding author for any interested researchers.)

### Statistical analysis

Before performing analyses for group comparisons, the Levene test was used to assess the homogeneity of variance. Descriptive statistics and *t*-tests were used to compare age according to gender. As the Levene test revealed a significant difference in DBS scores between genders, Welch’s *t*-test was used to compare the DBS scores on the basis of gender. A one-way analysis of variance was performed to assess the differences in DBS scores according to age. The significance level was set at *P* < 0.05.

To assess the factor structure of the Japanese version of the DBS, factor analysis (principal components, varimax rotation) was employed. Factor analysis uses the correlation matrix between items on a scale to determine whether a subset of items is related in such a way as to suggest that they are measuring the general concept of interest. The principal components method extracts factors and retains the maximum amount of common variance possible in the first factor, while subsequent factors keep the maximum amount of the remaining common variance. Factors are always listed in descending order according to the amount of variation they explain; i.e., from the highest (first) factor to the lowest (last) factor. An eigenvalue indicates the amount of variance explained by each factor, and eigenvalues above 1.0 are considered strong enough to be retained. Within each factor, item loading was categorized as follows: > 0.70 excellent, > 0.63 very good, > 0.55 good, and > 0.45 fair [19]. The highest loading in a factor was taken into account for each item. The Kaiser-Meyer-Olkin measure (KMO) was used to determine sampling adequacy. A KMO value equal to 0.70 indicates that factor analysis can be performed [19].

Cronbach’s alpha was used to test internal consistency. Reflecting the average intercorrelations of the items with each scale, Cronbach’s alpha has been high in previous studies that used the DBS [20, 21]. We chose to use Cronbach’s alpha as a measure of reliability for subscales based on the results of the factor analysis. All analyses were performed using a statistical software package (IBM SPSS Statistics, version 21, IBM Japan, Tokyo, Japan).

## Results

### DBS score by age and gender

The mean age of all participants was 14.1 ± 0.8 years. Girls comprised 42.1% of participants (mean age: 14.1 ± 0.8 years), while boys constituted 57.9% (mean age: 14.0 ± 0.8 years). No significant difference was found in age between girls and boys.

The total score of the Japanese-language DBS ranged from 15 to 75, with a mean value of 21.3 ± 10.5. The total mean score was 20.3 ± 9.1 for girls, and 21.9 ± 11.3 for boys. A significant difference among genders was observed in the DBS scores (*P* = 0.018), suggesting that boys exhibit greater negative perceptions toward the behavior of dentists than girls. However, no significant difference among ages was found in the DBS scores (*P* = 0.65). The highest ranked items, in descending order, were: “don’t feel comfortable asking questions,” “don’t feel I can stop for a rest,” “make me feel guilty,” and “worry if dentists are technically competent.” However, the ranking of these items differed between girls and boys. The means and standard deviations in the DBS of girls, boys, and all participants are shown in Table 3.

**Table 3:** DBS mean item scores and standard deviation (SD) for all adolescents (girls and boys)

Item	Girls (*N* = 403)	Boys (*N* = 554)	All (*N* = 957)
	Mean (SD)	Mean (SD)	Mean (SD)
1. Dentists don’t like requests	1.2 (0.6)	1.3 (0.8)	1.3 (0.7)
2. Dentists don’t have enough time	1.3 (0.8)	1.3 (0.8)	1.3 (0.8)
3. No clear explanations	1.3 (0.7)	1.4 (0.8)	1.4 (0.8)
4. Dentists don’t really listen	1.2 (0.7)	1.3 (0.9)	1.3 (0.8)
5. Do what they want, no matter what	1.4 (0.8)	1.5 (1.0)	1.5 (1.0)
6. Make me feel guilty	1.5 (1.0)	1.7 (1.1)	1.6 (1.0)
7. Not sure to believe what dentist says	1.2 (0.7)	1.4 (0.9)	1.3 (0.8)
8. Say things to try and fool me	1.1 (0.6)	1.3 (0.8)	1.2 (0.7)
9. Don’t take my worries seriously	1.2 (0.6)	1.3 (0.9)	1.3 (0.8)
10. Dentists make light of my fears	1.3 (0.8)	1.4 (1.0)	1.3 (0.9)
11. Worry if dentists are technically competent	1.5 (1.0)	1.6 (1.1)	1.5 (1.0)
12. Don’t think the dentist will stop if it hurts	1.4 (0.9)	1.5 (1.1)	1.4 (1.0)
13. Don’t feel I can stop for a rest	1.6 (1.1)	1.7 (1.3)	1.6 (1.2)
14. Don’t feel comfortable asking questions	1.7 (1.2)	1.7 (1.2)	1.7 (1.2)
15. Hearing news keeps me avoiding treatment	1.5 (1.0)	1.5 (1.0)	1.5 (1.0)
Total	20.3 (9.1)	21.9 (11.3)	21.3 (10.5)

### Factor analysis in all participants

The KMO value was 0.96, and Cronbach’s alpha for all 15 items was 0.95. The factor analysis yielded two factors with eigenvalues above 1.0, which collectively accounted for 65.8% of the variance. We labelled these factors as follows: Factor 1, “trust,” which corresponded to items 1, 4, 5, 7, 8, 9, and 10; and Factor 2, “lack of control,” which corresponded to items 11, 12, 13, 14, and 15 (Table 4). Cronbach’s alpha was 0.92 for Factor 1 and 0.88 for Factor 2, which suggests that these factors have good reliability.

**Table 4:** Rotated DBS factor matrix for all the adolescents (*n* = 957)

Item	Factor 1	Factor 2
1. Dentists don’t like requests	0.41*	0.12
2. Dentists don’t have enough time	0.25	0.23
3. No clear explanations	0.29	0.32
4. Dentists don’t really listen	0.55*	0.20
5. Do what they want, no matter what	0.53*	0.10
6. Make me feel guilty	0.36	0.34
7. Not sure to believe what dentist says	0.60*	0.36
8. Say things to try and fool me	0.75*	0.29
9. Don’t take my worries seriously	0.73*	0.28
10. Dentists make light of my fears	0.62*	0.41
11. Worry if dentists are technically competent	0.31	0.59*
12. Don’t think the dentist will stop if it hurts	0.41	0.77*
13. Don’t feel I can stop for a rest	0.29	0.81*
14. Don’t feel comfortable asking questions	−0.02	0.61*
15. Hearing news keeps me avoiding treatment	0.25	0.47*
Eigen value	8.8	1.0
% of explained variance	58.9	6.8
Cumulative %	58.9	65.8

* The highest loading (above 0.4) for each item is formatted in bold

### Factor analysis in girls and boys

The KMO value was 0.93 for girls and 0.96 for boys, and the factor structure differed according to gender. Thus, by including only factors with eigenvalues above 1.0, the DBS for girls comprised two factors: Factor 1, “trust” (seven items); and Factor 2, “communication” (three items) (Table 5). The DBS for boys comprised two different factors: Factor 1, “lack of control” (six items); and Factor 2, “belittlement” (six items) (Table 6).

**Table 5:** Rotated DBS factor matrix for girls (*n* = 403)

Item	Factor 1	Factor 2
1. Dentists don’t like requests	0.34	0.72*
2. Dentists don’t have enough time	0.29	0.79*
3. No clear explanations	0.20	0.62*
4. Dentists don’t really listen	0.56*	0.52
5. Do what they want, no matter what	0.57*	0.39
6. Make me feel guilty	0.58*	0.10
7. Not sure to believe what dentist says	0.62*	0.35
8. Say things to try and fool me	0.85*	0.25
9. Don’t take my worries seriously	0.81*	0.30
10. Dentists make light of my fears	0.59*	0.49
11. Worry if dentists are technically competent	0.34	0.20
12. Don’t think the dentist will stop if it hurts	0.29	0.24
13. Don’t feel I can stop for a rest	0.25	0.17
14. Don’t feel comfortable asking questions	−0.02	0.33
15. Hearing news keeps me avoiding treatment	0.34	0.12
Eigen value	8.3	1.2
% of explained variance	55.5	8.2
Cumulative %	55.5	63.7

* The highest loading (above 0.4) for each item is formatted in bold

**Table 6:** Rotated DBS factor matrix for boys (*n* = 554)

Item	Factor 1	Factor 2
1. Dentists don’t like requests	0.16	0.48*
2. Dentists don’t have enough time	0.29	0.24
3. No clear explanations	0.36	0.30
4. Dentists don’t really listen	0.30	0.49*
5. Do what they want, no matter what	0.26	0.27
6. Make me feel guilty	0.56*	0.18
7. Not sure to believe what dentist says	0.44	0.61*
8. Say things to try and fool me	0.30	0.73*
9. Don’t take my worries seriously	0.32	0.73*
10. Dentists make light of my fears	0.52	0.64*
11. Worry if dentists are technically competent	0.66*	0.29
12. Don’t think the dentist will stop if it hurts	0.71*	0.41
13. Don’t feel I can stop for a rest	0.81*	0.23
14. Don’t feel comfortable asking questions	0.75*	0.20
15. Hearing news keeps me avoiding treatment	0.60*	0.41
Eigen value	9.1	1.0
% of explained variance	60.9	6.4
Cumulative %	60.9	67.3

* The highest loading (above 0.4) for each item is formatted in bold

## Discussion

In this study, we obtained the mean DBS score of 21.3 ± 10.5. The score we obtained for Japanese adolescents was lower than those for Swedish [22], Singaporean [1], and Norwegian [5, 6] adolescents. Klingberg et al. [23] have argued that particular cultural and social habits along with the dental care system may affect the development of dental fear in adolescents. Regarding the dental care system in Japan, annual oral checkups for junior high school students are conducted as per the School Health and Safety Act. Dentists employed by the school conduct oral examinations, which include checking for dental/periodontal conditions and existence of temporomandibular disorder symptoms, malocclusion, and dental plaque/calculus by using dental mirrors under artificial light. These examinations are non-invasive and of a shorter duration than actual dental treatments. This regular event may contribute to the comparative low DBS score. In terms of cultural habits, we did not examine the cultural relevance of the DBS questions for the Japanese adolescent sample. Generally, Japanese men are encouraged to remain silent about their emotions, which is a cultural habit that could contribute to our findings, since the DBS asks them about fear and anxiety, and this may be a study limitation. However, in the present study, boys exhibited significantly higher DBS scores than girls. Thus, we consider that the questions were appropriate for the Japanese cultural context and did not need modification.

Given the narrow age range (13–15 years) in our study population, the relationship between age and level of dental fear was not observed. As past studies have indicated that younger patients are more anxious than older patients [9, 24], further study using broader age brackets is needed to confirm the correlation between age and the DBS scores. However, previous studies have also reported no significant differences between the DBS scores of female and male participants [5, 21, 22, 25]. Only one study, on 13- to 15-year-old participants in a population-based sample, showed that boys reported higher DBS scores than girls [1]. Moreover, the DBS has been found to correlate positively with other fear scales—such as the Dental Anxiety Scale and the Dental Fear Survey [7, 21, 26]. In general populations, levels of dental anxiety were significantly higher in women compared to men [9, 10]; thus, we expected that the DBS score would be higher in girls than boys. Contrary to this expectation, boys demonstrated a slightly but significantly higher DBS score than girls in our study.

It has been found that participants who have been patients for many years may demonstrate positive attitudes toward their dentist, as well as a higher degree of satisfaction with style of treatment [21]. In our study, 62 boys (11.2% of the total number of boys) and 30 girls (7.4% of the total number of girls) had never visited a dental clinic (data not shown). However, we could not attempt to investigate the routine dental visits of the participants, so further research is necessary in this respect. A larger sample composed of participants who routinely visit the dentist and who never visit the dentist would enable such analysis regarding the degree of confidence in dentists.

The number of items included in the factor structure was nearly identical to the original version of the DBS. In our study, only two dimensions were identified. Factor 1 consisted of seven items—1, 4, 5, 7, 8, 9, and 10—which we labelled “trust” based on the content of items 7 and 8. Factor 2 consisted of five items—11, 12, 13, 14, and 15—which we labelled “lack of control” based on the content of items 12 and 13. These were intended to capture two distinct dimensions concerning patient attitudes and perceptions toward dental care among Japanese adolescents.

Interestingly, gender difference was not limited to the DBS score—the factor structure also showed the gender difference. Dental anxiety can be acquired after feeling a lack of control in the dental treatment situation [22]. The perception of having control over aversive stimuli has been shown to reduce the stressfulness of the event, while diminished feelings of control increase agitation during stressful situations [1]. Indeed, a study reported that children who perceived a lack of control at the dentist were 13.7 times more likely to report high fear and 15.9 times less likely to return to the dentist willingly [1]. In our study, the boys assumed that “lack of control” and “belittlement” were the most important factors for confidence in dentists. In contrast, the girls assumed that “trust” and “communication” were the most important factors, while “lack of control” was excluded.

Lu et al. [27] described that Asian children may develop a greater fear of pain as a result of Asian parents guarding them from challenging situations. Moreover, empirical evidence suggests that despite their desire to express emotions, Asians are less emotionally expressive than Caucasians [28]. These tendencies may hinder communication or cause a lack of control over aversive stimuli at the dental clinic. Indeed, the cultural background in Japan—where it is considered a virtue for men to remain silent in such circumstances—may impact this situation. Women are more likely to express their fears, whereas men may not express their fears as openly as women [24]. Therefore, boys may feel they do not have control over aversive stimuli at the dental clinic. These factors may shape the gender difference in factor structure.

Hence, for boys, strategies to enhance the patient’s sense of control—such as making the patient more active in the treatment, signaling the beginning and end of procedures, and providing the opportunity to ask questions—are recommended in the dental environment [4]. In contrast, strategies that enhance the impression of sympathy for patients—such as listening to their concerns, explaining procedures thoroughly, and increasing their feeling of choice—are recommended for girls.

Despite our findings, this study does have a limitation. The DBS has been revised and expanded to a 28-item version (the Revised DBS, R-DBS), reflecting increased understanding of the concerns of fearful patients [29]. Additionally, the R-DBS has been translated into a number of languages, and different language versions have been created [30–32]. However, we used the original DBS in this study because the R-DBS has many more questionnaire items than the original and would take more time to complete. As the present study was conducted with another survey regarding temporomandibular disorders, the questionnaire items were limited so participants could answer all items in the allotted time. Consequently, we cannot directly compare the findings of the present study, which uses the DBS score, to other studies using the R-DBS score. A future study using a Japanese translation of the R-DBS is needed to assess gender differences and compare Japanese participants’ R-DBS scores with those of other populations.

## Conclusions

This study identified the mean value of the scores of the Japanese-language DBS, as well as two factors of differing strengths pertaining to Japanese adolescents’ confidence in dentists. Gender differences in perceptions toward dentists were also observed: boys regard “lack of control” and “belittlement” as important factors for confidence in dentists, whereas girls require “trust” and “communication.” As suggested in this paper, accounting for these differences may improve the effectiveness of strategies intended to lower dental anxiety and foster positive dental beliefs in young patients.

## Data Availability

The datasets used and/or analyzed during this study are available from the corresponding author upon reasonable request.

## Abbreviations

DBS: Dental beliefs survey
ICC: Intraclass correlation coefficient
KMO: Kaiser-Meyer-Olkin measure
R-DBS: Revised dental beliefs survey
